# Replicative Senescence in Human Fibroblasts Is Delayed by Hydrogen Sulfide in a *NAMPT/SIRT1* Dependent Manner

**DOI:** 10.1371/journal.pone.0164710

**Published:** 2016-10-12

**Authors:** Reiko Sanokawa-Akakura, Shin Akakura, Siamak Tabibzadeh

**Affiliations:** Frontiers in Bioscience Research Institute in Aging and Cancer, Irvine, CA, United States of America; Tulane University Health Sciences Center, UNITED STATES

## Abstract

Recent evidence suggests that hydrogen sulfide (H_2_S) has cytoprotective and anti-aging effects. However, the mechanisms for such properties are not fully understood. Here, we show that the expression of the main H_2_S producing enzyme, *CBS*, and production of H_2_S are coordinately diminished in replicative senescent adult human dermal fibroblasts. The reduced production of H_2_S falls within the same time-frame that the hallmarks of replicative senescence appear including accumulation of SA–β-Gal, enhanced expression of *p16*, *p21*, and *RRM2B* while the expression of *RRM2*, *hTERT*, *SIRT1*, *NAMPT*, and NAD/NADH ratio all fall. Exogenous H_2_S increases the expression of *hTERT*, *NAMPT*, *SIRT1* and NAD/NADH ratio in treated cells. Moreover, H_2_S safeguards the expression of *hTERT* in a *NAMPT* and *SIRT1* dependent manner and delays the onset of replicative senescence as evidenced by reduced accumulation of age associated SA–β-Gal and cessation of proliferation. Postponement of loss of cell proliferative capacity without risk of mutagenesis shows implications for use of H_2_S in delaying the adverse effects of senescence in organisms.

## Introduction

There are several lines of evidence that the gasotransmitter, H_2_S has cytoprotective and life extension properties. Recently, it was shown that the generation of reactive oxygen species is increased in knockouts of mpst-1, a major enzyme that drives the production of hydrogen sulfide in *C*. *elegans* and this deficit is overcome by the administration of GY4137 that exposes the short-lived mutants to hydrogen sulfide [1]. This treatment also extends the lifespan of normal animals and delays the onset of detrimental impact of senescence as assessed by pharyngeal contraction and defecation [1]. The extension of lifespan by hydrogen sulfide, which requires SIR-2.1 activity, affords the animals other health-promoting effects including stress resistance and improved thermotolerance [2].

It is known that calorie restriction promotes longevity by increasing *SIRT1* expression [3]. In yeast and *Drosophila*, calorie restriction extends life-span by increasing Sir2 activity and by activating Sir2 deacetylase. Senescence is thought to be due to a progressive loss of cell function and/or cell loss over time. *SIRT1* reduces stress induced apoptotic cell loss by deacetylation of the DNA repair factor, Ku70. Deacetylated Ku70, in turn, reduces apoptosis by sequestering the proapoptotic factor, Bax, away from the mitochondria. Thus, by inducing *SIRT1* expression, calorie restriction promotes long-term survival of cells which are irreplaceable [3]. It was recently shown that the effect of calorie restriction on life extension is associated with an increase in production of hydrogen sulfide with a cysteine and methionine deficient diet being required for such an enhanced production [4].

In light of such evidence, here, we tested the hypothesis that replicative senescence is associated with a progressive loss in ability of cells to produce hydrogen sulfide and that supporting fibroblasts with an exogenous source of hydrogen sulfide delays replicative senescence that ultimately leads to cessation of proliferation. Data shown here support the view that the life extension properties of hydrogen sulfide, at least in part, is due to its impact in safeguarding against senescence in a *NAMPT* and *SIRT1* dependent manner.

## Materials and Methods

### Reagents and cell culture

Cell viability was confirmed by Trypan Blue staining (Sigma-Aldrich, St Louis, MO). Chemicals were from Sigma-Aldrich, TRIZOL^®^ and reverse transcriptase (RevertAid^®^ Reverse Transcriptase) were from Thermo Scientific (Carlsbad, CA). *NAMPT* siRNA was purchased from Santa Cruz Biotechnology (Dallas, TX). Oligonucleotides were generated by IDT (Coralville, IA). Transfection reagent was purchased from Santa Cruz Biotechnology. Adult human dermal fibroblasts (aHDF) cells were obtained from ATCC (Manassas, VA) or Lonza (Walkersville, MD). Cells were maintained in Fibroblast Growth Medium (FGM, Lonza) with 2% fetal bovine serum and growth factors in a 37°C incubator with 5% CO_2_.

### Determination of Population Doublings (PD)

Culture dishes were seeded in triplicates with 3 x 10^5^ aHDF cells. We calculated PD based on the following formula: log ((number of cells harvested)/(number of cells seeded))/log2 + previous PD. Since the PD of cells received from manufacturer was not known, we defined the first PD after initial culture as 0 [5].

### Staining for Senescence-Associated β-Galactosidase (SA-β-Gal)

SA–*β*-Gal staining was performed as described previously [6].

### Measurement of H_2_S production

H_2_S production was measured by WPI instrument as described previously (Sarasota, FL) [7,8].

### Real-time PCR

Real-time PCR was performed using iTaq^®^ Universal SYBR^®^ Green Supermix (Bio-Rad; Hercules, CA) and LightCycler^®^ 96 (Roche Diagnostics, Indianapolis, IN) according to the manufacturer’s instruction. Primers were purchased from IDT (S1 Table).

### Immunoblotting

Immunoblotting is described previously [8]. 10 μg of total protein lysates for Nampt and β Actin and 30 μg of total protein lysates for Sirt1 were used. For hTERT blotting, nuclear extraction was performed [9] and 100 μg of nuclear extracted lysates were used. Used antibodies are; anti-hTERT mouse monoclonal (clone 2C4; EMD Millipore), anti-Nampt mouse monoclonal (Sigma), anti-Sirt1 rabbit polyclonal (Sigma), and anti-β Actin-HRP (sc1616-HRP; Santa Cruz). The substrate used in this study was ECL^®^ Prime Western Blotting Detection Reagent (GE Healthcare). The membranes were scanned using C-Digit^®^ (LI-COR) and analyzed by Image Studio^®^ (LI-COR).

### Telomerase activity assay

Cells were harvested and lysed in CHAPS buffer (0.5% CHAPS, 10 mM Tris-HCl, pH = 7.5., with 1.5. mM MgCl_2_, 1 mM EGTA, 10% glycerol). The telomeric repeat amplification protocol (TRAP) assay was performed as described by Kim *et al*. [10,11]. Briefly, PCR was performed using primers listed in S1 Table as follows: first incubation at 30°C for 30 min, second incubation at 95°C for 3 min, followed by a 30 cycle amplification (95°C for 30 s, 59°C for 30 s, and 72°C for 1 min). The products were run on a 15% polyacrylamide gel (Bio-Rad) in 0.5x TBS and the bands were stained with SYBR Gold Nucleic Acid Gel Stain (Life Technologies). Relative activity of telomerase was calculated by dividing the density of the all ladders to the density of the bands in internal control (The TRAP internal control “TSNT” was synthesized as reported previously (S1 Table) [11]. Densitometric analysis was performed using ImageJ (NIH, Bethesda, MD).

### NAD assay

NAD assay was performed using EnzyChrom^®^ NAD^+^/NADH^+^ Assay kit (BioAssay Systems, Hayward, CA), according to the manufacturer’s instruction.

### siRNA transfection

*NAMPT* siRNA and Scrambled siRNAs (control siRNAs) were purchased from Santa Cruz Biotechnology. *SIRT1* siRNA and oligonucleotides were generated by IDT. The sequence of *SIRT1* siRNA was described previously [12]. Each oligonucleotide was dissolved in 100 μM Duplex Buffer (100 mM Potassium Acetate, 30 mM HEPES, pH 7.5) and mixed in equal molar amounts, with a final concentration of 10 μM per oligonucleotide. Oligonucleotides were annealed at 94°C for 2 minutes and then cooled to room temperature for 2 hours. Transfection of siRNA into aHDF cells was carried out at 37°C and with 5% CO_2_ for 2 days using transfection reagent (Santa Cruz Biotechnology) in DMEM medium without serum and antibiotics. The culture medium was replaced with fibroblast medium (FGM2, Lonza, Walkersville, MD) without or with 1 μM NaHS and cells were cultured for an additional 3 days.

### SIRT1 activity assay

SIRT1 activity assay was performed using Universal SIRT Activity Assay Kit (Abcam, Cambridge, MA) according to the manufacturer’s instruction. Briefly, 5 x 10^5^ of aHDF cells were transfected with *NAMPT* siRNA or Scrambled-siRNA. After 2 days, fresh medium was added without or with 1 μM NaHS and cells were cultured for an additional 3 days. Sample cells were collected and nuclear fractions were prepared, as described previously [13]. SIRT1 activity was normalized to total protein of each sample.

### Statistics

All assays were done in 3–6 replicate cultures, in at least three independent experiments. Data are shown as means ± SEM. *p* values were determined by comparing the data from treated cells against control cells. Data were subjected to the two tailed t-test for determination of means and *p* values. *p* values less than 0.05 were considered significant. *p* values are shown as <0.05 (*), <0.005 (**) or <0.0005 (***).

## Results

### Replicative senescence leads to reduced production of hydrogen sulfide

**Fig 1:**
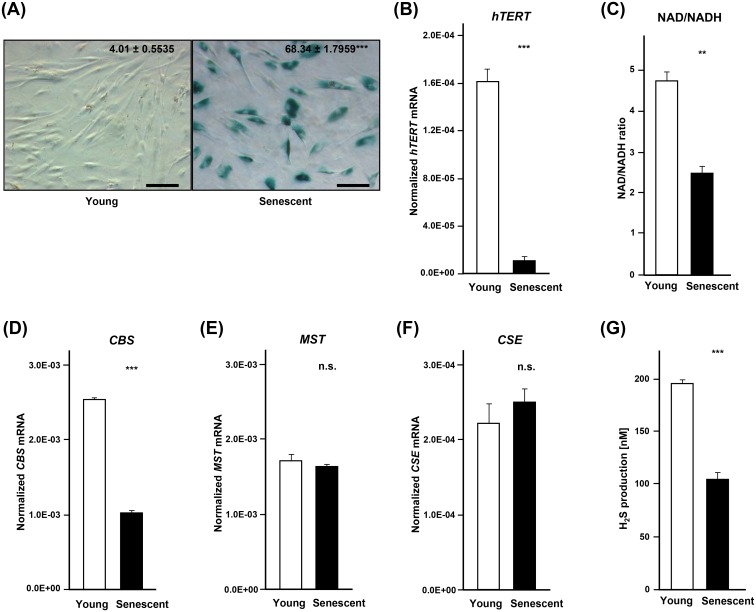
Production of H_2_S is downregulated in replicatively senescent cells. (**A**) Representative images of SA-β-Gal staining in young (PD: 5.9) and senescent (PD: 18.8) aHDF cells. Scale bars, 100 μm. (**B**) Real-time PCR analysis of expression of *hTERT* in young (PD: 5.9) and senescent (PD: 18.8) aHDF cells. The expression of *hTERT* was normalized to the expression level of *β-ACTIN*. (**C**) NAD/NADH ratio in young (PD: 5.9) and senescent (PD: 18.8) aHDF cells. Real-time PCR analysis of expression of *CBS* (**D**), *MST* (**E**), and *CSE* (**F**) in young (PD: 5.9) and senescent (PD: 18.8) aHDF cells. The expression of *CBS*, *MST*, and *CSE* was normalized to the expression level of *β-ACTIN*. (**G**) 1 x 10^6^ cells of young (PD: 5.9) and senescent (PD: 18.8) aHDF cells were incubated in PBS at 37°C for 1 hour and then H_2_S was measured in culture supernatants. Mean values are shown along with error bars. *; *p*<0.05, **; *p*<0.005, ***; *p*<0.0005, n.s.; not significant.

We assessed the production of H_2_S in young (population doubling; PD: 5.9) and replicative senescent (PD: 18.8) aHDF cells. Consistent with previous reports, senescent cells show accumulation of senescence-associated β-galactosidase (SA–β-Gal) (68%, Fig 1A), increase in expression of *p16*, *p21*, *RRM2B* and decreased expression of *RRM2* [14] (S1 Fig). The expression *hTERT* was also diminished by about 16 fold in senescent aHDF cells (Fig 1B). As compared to young cells, the NAD/NADH ratio which is a measure of metabolic activity was also decreased in senescent cells (Fig 1C).

Then, we assessed whether the expression of three H_2_S-producing enzymes, *CBS*, *MST* and *CSE* [15–17] is altered by senescence. The expression of *CBS* was decreased in senescent cells while the expression of *MST* and *CSE* remained the same in young and replicatively senescent cells (Fig 1D–1F). The production level of H_2_S in replicative senescent cells was diminished by 63% in senescent cells (Fig 1G).

### Exogenous H_2_S upregulates the expression of *hTERT* and increases PD in aHDF cells

**Fig 2:**
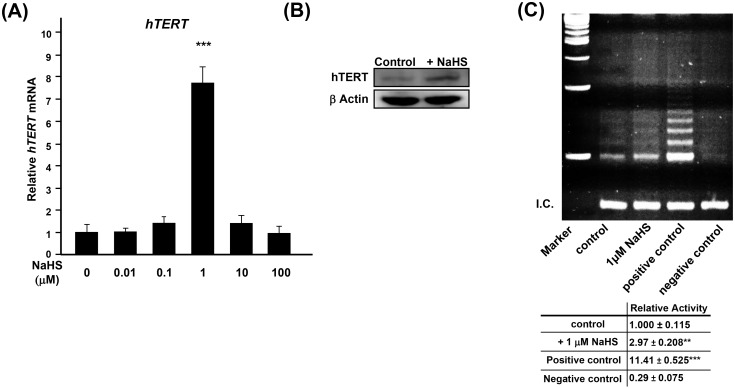
Exogenous H_2_S increases the expression of *hTERT* as well as the activity of telomerase. (**A**) Real-time PCR analysis of the expression of *hTERT* in young (PD: 5.9) aHDF cells, treated with NaHS for 3 days. The expression of *hTERT* was normalized to the level of expression of *β-ACTIN*. Expression of untreated control was regarded as 1.0. (**B**) Immunoblotting of hTERT in aHDF cells without or with 1 μM NaHS for 7 days. 100 μg of the indicated nuclear extracts were subjected for immunoblotting. β-Actin was used as a loading control. (**C**) Telomerase activity in young (PD: 3.2) aHDF cells without or with treated with 1 μM NaHS for 7 days. Positive control was MDA-MB-231 cell lysate, and negative control was buffer alone. Bottom panel shows quantified means ± error bars from three independent assays. Relative activity of telomerase was calculated by dividing the density of all ladders to the density of the bands in internal control, indicated as internal control (I.C.).

We tested whether the lower expression of *hTERT* in senescent aHDF cells is due to down-regulation of H_2_S production in senescent cells. Treatment of young aHDF cells with NaHS within the reported physiological level of H_2_S [18] (0.01 to 100 μM) significantly upregulated the expression of *hTERT* with 1 μM being the optimal concentration for maximum *hTERT* expression (Fig 2A). This finding was confirmed by using other cell types (S2A–S2C Fig), and as evidenced by immunoblotting (NaHS enhanced hTERT in treated cells; Fig 2B). However, treatment with NaHS failed to reduce SA-β-Gal or to upregulate the expression of *hTERT* in senescent aHDF cells (~65% SA-β-Gal^+^) (S3A and S3B Fig), suggesting that H_2_S induced *hTERT* expression is suppressed in senescent cells.

**Fig 3:**
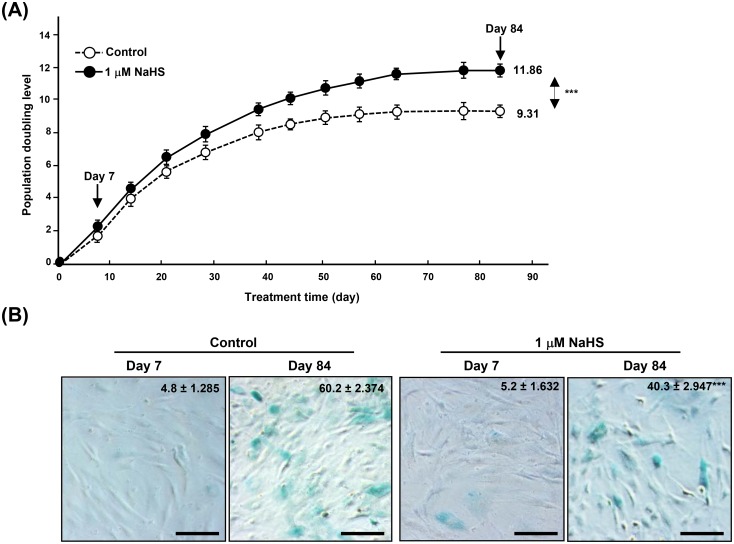
Exogenous H_2_S increases PD and suppresses SA-β-Gal expression. (**A**) PD of cells treated without or with 1 μM NaHS. The population doubling of the first confluent cultures was designated as 0. (**B**) Representative images of SA–β-Gal staining in cells shown in Fig 3A. Mean values ± error bars of number of SA-β-Gal positive cells are shown on the right-upper corner of each image. *; p<0.05, ***; p<0.0005, n.s.; not significant. Scale bars, 100 μm.

Up-regulation of expression of *hTERT* in young aHDF cells treated with NaHS was associated with an increase in the activity of telomerase (Fig 2C). We, then, investigated whether the increased expression of *hTERT* and telomerase activity increases PD, young aHDF cells were treated weekly with 0, 1, and 100 μM of NaHS for 84 days, and PD was calculated. As shown in Fig 3A, as compared to PD of the untreated control groups, treatment of aHDF cells with 1 μM NaHS caused a significant increase in PD. This increase was lost in cells that were treated with greater (100 μM) concentration of NaHS (S4 Fig). Consistent with these data, the percentage of SA-β-Gal positive cells was reduced in aHDF cells that were treated with 1 μM of NaHS (Fig 3B).

### H_2_S mediated *hTERT* expression is *NAMPT* and *SIRT1* dependent

**Fig 4:**
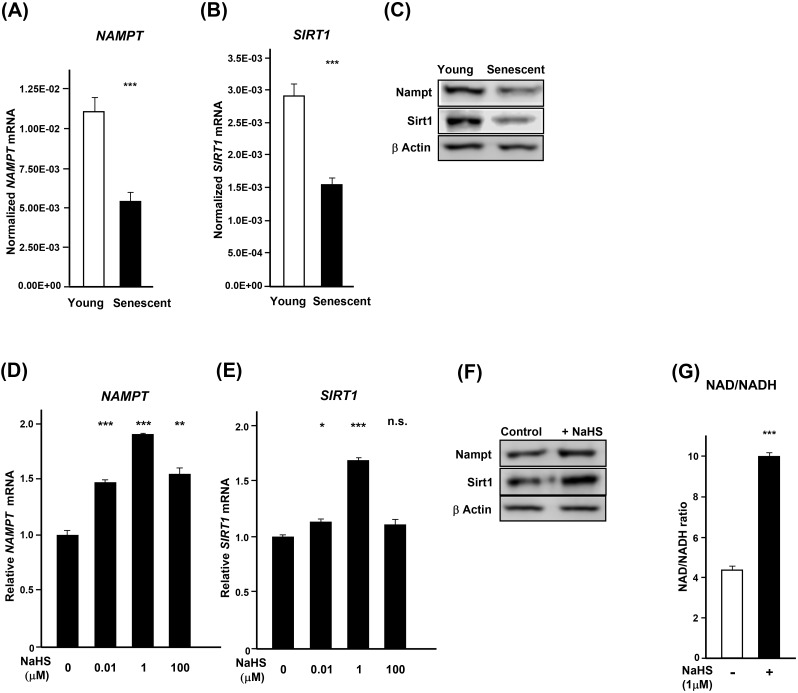
NaHS-treatment increases expression of *NAMPT* and *SIRT1*. (**A** and **B**) The expression of *NAMPT* and *SIRT1* in young (PD: 5.9) and senescent (PD: 18.8) was assessed by real-time PCR and normalized to the expression level of *β-ACTIN*. (**C**) Immunoblotting of Nampt and Sirt1 in young (PD: 5.9) and senescent (PD: 18.8) aHDF cells. β-Actin was used as a loading control. (**D** and **E**) Young (PD: 5.9) aHDF cells were treated without and with NaHS for 3 days, and RNA samples were then subjected to real-time PCR for assessment of *NAMPT* and *SIRT1*. The expression levels of *NAMPT* and *SIRT1* were normalized to the levels of expression of *β-ACTIN*. (**F**) Immunoblotting of Nampt and Sirt1 in NaHS-treated young (PD: 5.9) aHDF cells. β-Actin was used as a loading control. (**G**) NAD/NADH ratio in young (PD: 5.9) aHDF cells treated without and with NaHS for 7 days. Data were normalized to the total amount of protein.

**Fig 5:**
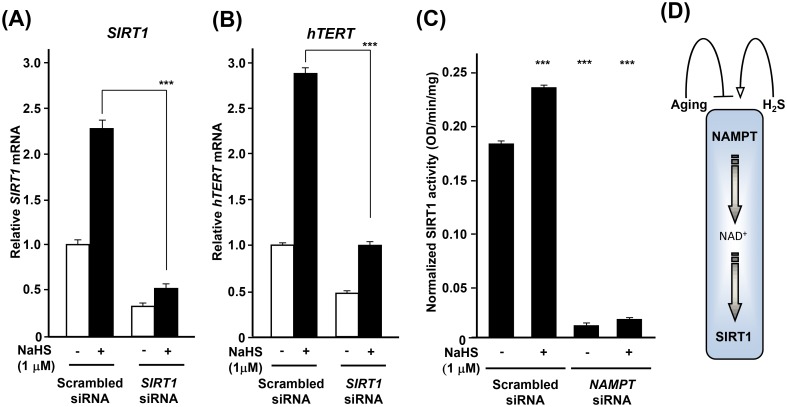
H_2_S induces *hTERT* expression in a NAMPT/SIRT1-dependent manner. (**A** and **B**) Downregulation of *SIRT1* suppresses the expression of *hTERT*. Young (PD: 5.9) aHDF cells (3 x 10^5^ cells) were transfected with *SIRT1* siRNA for 2 days, and these were treated without or with NaHS for 3 days. Total RNAs from these cells were subjected to real-time PCR analysis for *SIRT1* (**A**) and *hTERT* (**B**). Data were normalized to the level of expression of *β-ACTIN*. The expression level of *SIRT1* and *hTERT* in cells treated with Scrambled siRNA without NaHS treatment was regarded as 1.0. (**C**) Downregulation of *NAMPT* suppresses the activity of SIRT1. Young (PD: 5.9) aHDF cells (3 x 10^5^ cells) were transfected with *NAMPT* siRNA for 2 days, and then the cells were treated without or with 1 μM NaHS for 3 days. Nuclear proteins were extracted and used for measurement of SIRT1 activity. Mean values ± error bars were normalized to the amount of total cell protein. (**D**) Mode of action of H_2_S in opposing senescence. *; *p*<0.05, **; *p*<0.005, ***; *p*<0.0005, n.s.; not significant.

The life extension afforded by hydrogen sulfide [2,19,20] might be mediated, at least in part, by increasing the expression of *NAMPT* (nicotinamide phosphoribosyl transferase) that regulates metabolism together with *SIRT1*, a factor involved in the maintenance of integrity of telomeres [21]. Among the seven Sir2 homologues in mammalian cells (*SIRT1* to *-7*), SIRT1 is most closely related to Sir2 which is known to be a major life-span regulator in *C*. *elegans* [2]. Nicotinamide adenine dinucleotide (NAD^+^) is a coenzyme that mediates many redox reactions and regulates NAD^+^-consuming enzymes such as Sirtuin family of NAD—dependent protein deacetylases. The biosynthesis of NAD^+^ is mediated by NAMPT. For these reasons, we examined whether the effect of H_2_S on *hTERT* is *NAMPT* and *SIRT1* dependent. In senescent aHDF cells, the expression of *NAMPT* and *SIRT1* diminished with age (Fig 4A–4C). Treatment of young aHDF cells (PD: 5.9) with NaHS increased the expression of *NAMPT* and *SIRT1*, in a dose dependent manner with 1 μM inducing maximum expression (Fig 4D and 4E). These data were further verified by immunoblotting (Fig 4F). The treatment also caused a coordinate increase in the ratio of NAD to NADH (Fig 4G). Treatment of aHDF cells with *SIRT1* siRNA suppressed the NaHS induced expression of *SIRT1* (Fig 5A and S5 Fig) and concomitantly prevented the expression of *hTERT* (Fig 5B). Whereas the siRNA to *NAMPT* reduced expression of *NAMPT*, it did not reduce the expression of *SIRT1* (S5 Fig). However, suppression of *NAMPT* decreased the activity of SIRT1 (Fig 5C) and led to a decrease in the expression of *hTERT* (S5 Fig).

## Discussion

We demonstrated that the production of H_2_S as well as the expression of *CBS* both decrease upon aging. We further show that the downregulation of *hTERT*, *SIRT1*, *NAMPT*, and NAD/NADH ratio can be delayed by H_2_S and that long-term effect of H_2_S is to maintain telomerase expression, and to postpone replicative senescence as evidenced by increasing population doublings in aHDF cells treated with exogenous H_2_S. Thus, H_2_S maintains a threshold level of telomerase activity which contributes to its life-span extension properties.

H_2_S plays a bioenergetics role in Krebs cycle in mitochondria [22]. Modis *et al* showed that low concentrations of H_2_S elicited an increase of mitochondrial function, including an increase cellular pool of ATP and improved cell viability, whereas higher concentrations of H_2_S were inhibitory [22,23]. Our data show 1 μM of NaHS is optimal for promoting *hTERT* expression (Fig 2A) and in increasing PD (Fig 3A and S4 Fig). Regardless of site of action, H_2_S leads to an increase in cellular pool of ATP energy yield which results in suppressing cellular senescence in aHDF cells.

Previously, we have shown that H_2_S upregulates NAMPT and increases mitochondrial bioenergetics [7,8]. Although it is still not clear how H_2_S controls NAMPT, Huang *et al* reported that H_2_S suppresses the expression of microRNA34a by activating Nrf2 after hepatic ischemia/reperfusion injury [24]. Choi *et al* demonstrated that microRNA34a reduced NAMPT/NAD+ level [25]. Based on such finding H_2_S might negatively regulate NAMPT by suppression of microRNA34a. However, further studies are required to address the molecular mechanisms of NAMPT by H_2_S.

Others have shown the relation of NAMPT, NAD and SIRT1 [26,27]. It has been shown that *SIRT1* by deacetylation of *c-MYC* [28] transcriptionally increases the activation of *c-MYC* and correspondingly increases the amount of acetylated H4 histone at the *hTERT* promoter [21]. In addition, *FOXO3a*, a downstream target of *SIRT1*, potentiated *hTERT* gene transcription by binding to *c-MYC* promoter. This upregulated *c-MYC* which was recruited to the *hTERT* promoter, leads to the of *hTERT* gene activation [29]. Intriguingly, NaHS-treatment increases the expression of *FOXO3a* in aHDF cells (data not shown); thus, it seems that *SIRT1* upregulates *hTERT* through *FOXO3a/c-MYC* and increases the lifespan of human fibroblasts.

Mammalian senescence is dependent on the mammalian NAD-dependent deacetylase, Sirt1, and Nampt-mediated systemic NAD biosynthesis [30]. Based on our findings, H_2_S regulates the expression of *NAMPT* and *SIRT1* in a dose dependent manner and coordinately sets the NAD/NADH ratio. H_2_S also regulates *hTERT* expression, and this function is dependent on both *NAMPT* mRNA expression and SIRT1 activity. Sir2 and its orthologues play an important role in controlling longevity in model organisms as diverse as yeast to worms and flies [31]. Among sirtuins, it has been shown that *SIRT1* delays senescence and extends life-span in both male and female mice [32]. In light of such findings, the life extension afforded by H_2_S might be mediated, at least in part, through activation of *SIRT1* (Fig 5D). Consistent with these results, the treatment of human umbilical vascular endothelial cells with H_2_S, delayed the H_2_O_2_ and nicotinamide induced pre-mature senescence by SIRT1 activation [33,34]. The impact of H_2_S on NAMPT/SIRT1, likely, has global effects since it has been shown that RNA-mediated knockdown of *NAMPT* or *NMNAT-1* in MCF-7 breast cancer cells reduced total cellular NAD^+^ levels and globally altered pattern of gene expression [35]. Together, the postponement of loss of cell proliferative capacity by H_2_S without the risk of mutagenesis suggests that H_2_S can be used in delaying the adverse effects of senescence in organisms.

## Supporting Information

S1 Fig Verification of senescence. (DOC)

S2 Fig Effect of NaHS-treatment on other human fibroblasts. (DOC)

S3 Fig Treatment of senescence cells with NaHS is not effective to suppress cellular senescence. (DOC)

S4 Fig Treatment of aHDF cells with 100 μM NaHS does not increase PD. (DOC)

S5 Fig Down regulation of *NAMPT* suppresses the expression of *hTERT*, but not *SIRT1*. (DOC)

S1 Table Primer sequences. (DOC)

## Abbreviations

H_2_S: hydrogen sulfide
aHDF: adult human dermal fibroblasts
SA-β-Gal: Senescence-Associated β-Galactosidase
*RRM2*: *ribonucleotide reductase M2*
*RRM2b*: *ribonucleotide reductase M2B*
*CSE*: cystathionine-β-lyase
*CBS*: cystathionine-*β*-synthase
*MST*: 3-mercaptopyruvate sulftransferase
*hTERT*: catalytic subunit of human telomerase
NaHS: sodium hydrosulfide
HRP: horseradish peroxidase
Ct: cycle threshold
*NAMPT*: Nicotinamide phosphoribosyltransferase
s: second
h: hour
PD: population doubling
