# Supplementary material for: Replicative Senescence in Human Fibroblasts Is Delayed by Hydrogen Sulfide in a NAMPT/SIRT1 Dependent Manner
Source: PLoS One. 2016 Oct 12;11(10):e0164710. doi: 10.1371/journal.pone.0164710 (PMC5061390; doi:10.1371/journal.pone.0164710)
Supplement: S1 Fig — (DOC) [file pone.0164710.s001.doc]

**S1 Fig. Verification of senescence.** Young (5.9 PD) and senescent (18.8 PD) aHDF cells were subjected for real-time PCR using *p16*- **(A)**, *p21*-**(B)**, *RRM2*-**(C)**, and *RRM2b*-**(D)** specific primers. The expression levels of *p16*, *p21*, *RRM2* and *RRM2b* were normalized to the level of expression of *β-ACTIN*. Mean values with error bars are shown. ***; *p*<0.0005.
