# Supplementary material for: Replicative Senescence in Human Fibroblasts Is Delayed by Hydrogen Sulfide in a NAMPT/SIRT1 Dependent Manner
Source: PLoS One. 2016 Oct 12;11(10):e0164710. doi: 10.1371/journal.pone.0164710 (PMC5061390; doi:10.1371/journal.pone.0164710)
Supplement: S2 Fig — (DOC) [file pone.0164710.s002.doc]

**S2 Fig. Effect of NaHS-treatment on other human fibroblasts.** Young (4.4 PD) neonatal human dermal fibroblast (nHDF) cells, BJ1 cells (2.4 PD), and IRM90 cells (2.8 PD) were treated with 1 µM NaHS for 7 days and samples were subjected for real-time PCR using *hTERT*-specific primers. The expression level of hTERT was normalized to the level of expression of *β-ACTIN*. Mean values with error bars are shown. ***; *p*<0.0005. Note that PD was calculated after the first confluent cells as PD 0.
