# Supplementary material for: Replicative Senescence in Human Fibroblasts Is Delayed by Hydrogen Sulfide in a NAMPT/SIRT1 Dependent Manner
Source: PLoS One. 2016 Oct 12;11(10):e0164710. doi: 10.1371/journal.pone.0164710 (PMC5061390; doi:10.1371/journal.pone.0164710)
Supplement: S3 Fig — (DOC) [file pone.0164710.s003.doc]

**S3 Fig. Treatment of senescent cells with NaHS is not effective to suppress cellular senescence.** Young (5.9 PD) and senescent (18.8 PD) aHDF cells were treated without or with 1 µM NaHS for 7 days. These cells were stained for SA-β-Gal **(A)** and the expression of *hTERT* was analyzed by real-time PCR **(B)**. The expression of *hTERT* was normalized to the level of expression of β*-ACTIN* with the level of mRNA of young untreated control cells regarded as 1.0. Mean values with error bars are shown. ***; *p*<0.0005. n.s.; not significant. Scale bars, 100 µm.
