# Supplementary material for: Replicative Senescence in Human Fibroblasts Is Delayed by Hydrogen Sulfide in a NAMPT/SIRT1 Dependent Manner
Source: PLoS One. 2016 Oct 12;11(10):e0164710. doi: 10.1371/journal.pone.0164710 (PMC5061390; doi:10.1371/journal.pone.0164710)
Supplement: S4 Fig — (DOC) [file pone.0164710.s004.doc]

**S4 Fig. Treatment of aHDF cells with 100 µM NaHS does not increase PD.** Culture dishes were seeded, in triplicates, with 3 x 10^5^ aHDF cells (2.9 PD), and these cells were treated without or with 100 µM NaHS weekly. The number of cells was counted using Trypan Blue and PD was calculated as described in text. Mean values with error bars are shown. n.s.; not significant.
