# Supplementary material for: Replicative Senescence in Human Fibroblasts Is Delayed by Hydrogen Sulfide in a NAMPT/SIRT1 Dependent Manner
Source: PLoS One. 2016 Oct 12;11(10):e0164710. doi: 10.1371/journal.pone.0164710 (PMC5061390; doi:10.1371/journal.pone.0164710)
Supplement: S5 Fig — (DOC) [file pone.0164710.s005.doc]

**S5 Fig. Down regulation of *NAMPT* suppresses the expression of *hTERT*, but not *SIRT1*.** Real-time PCR analysis was carried out for the expression of *NAMPT*, *SIRT1,* and *hTERT* in aHDF cells (3 x 10^5^, 5.9 PD) that were transfected with *NAMPT* siRNA for 2 days and then were treated without or with 1 µM NaHS for 3 days. The expression levels of *NAMPT* (A), *SIRT1* (B) and *hTERT* (C) were normalized to the expression level of *β-ACTIN.*  Relative levels of mRNA in groups treated with scrambled siRNA without NaHS treatment was regarded as 1.0. Mean values ± error bars. ***; *p*<0.0005, n.s.; no significant. (D) Representative image of immunoblotting of Nampt or Sirt1 in aHDF cells treated with the indicated siRNA. Young aHDF cells (7.6 PD) were transfected with scrambled siRNA, *NAMPT* siRNA, or *SIRT1* siRNA for 2 days, then treated without or with 1 µM NaHS for 3 days. Cells were lysed and lysates were subjected for immunoblotting. β-Actin was used as a loading control.
