# Supplementary material for: Replicative Senescence in Human Fibroblasts Is Delayed by Hydrogen Sulfide in a NAMPT/SIRT1 Dependent Manner
Source: PLoS One. 2016 Oct 12;11(10):e0164710. doi: 10.1371/journal.pone.0164710 (PMC5061390; doi:10.1371/journal.pone.0164710)
Supplement: S1 Table — (DOC) [file pone.0164710.s006.doc]

**S1 Table.** Primer sequences

| **Gene name** | **Forward 5’- 3’** | **Reverse 3’- 5’** |
| --- | --- | --- |
| **Real time** |  |  |
| *β-ACTIN* | TTG CCG ACA GGA TGC AGA AGG A | AGG TGG ACA GCG AGG CCA GGA T |
| *CBS* | TCA AGA GCA ACG ATG AGG AG | ATG TAG TTC CGC ACT GAG TC |
| *hTERT* | GCG GAA GAC AGT GGT GAA CT | ACC TGG AGT AGT CGC TCT GC |
| *MST* | CGC CGT GTC ACT GCT TGA T | CAC CTG GAA GCG CCG GGA TT |
| *CSE* | AGA AGG TGA TTG ACA TTG AAG G | CAA TAG GAG ATG GAA CTG CTC |
| *NAMPT* | ATC CTG TTC CAG GCT ATT CTG | CCC CAT ATT TTC TCA CAC GCA T |
| *p16* | CGG AGA GGG GGA GAA CAG AC | CCG TAA CTA TTC GGT GCG TTG |
| *p21* | CCG TCT CAG TGT TGA GCC TT | CCT GGA GCT GAG AGG GTA CT |
| *RRM2* | CCC GCT GTT TCT ATG GCT TC | CCC AGT CTG CCT TCT TCT TG |
| *RRM2b* | GAG GCT CGC TGT TTC TAT GG | ATC TGC TAT CCA TCG CAA GG |
| *SIRT1* | AAG TTG ACT GTG AAG CTG TAC G | TGC TAC TGG TCT TAC TTT GAG GG |
| **siRNA** |  |  |
| *SIRT1* | GCA ACA GCA rUCrUr UGC CrUG ArUrUr UGrU A | rUAC AAAr UCA GGC AAG ArUG CrUGr UrUG C |
| **Telomerase** |  |  |
| *ACX* | GCG CGG CTT ACC CTT ACC CTT ACC CTA ACC |  |
| *Internal control oligomer* | AAT CCG TCG AGC AGA GTT AAA AGG CCG AGA AGC GAT |  |
| *NT* | ATC GCT TCT CGG CCT TTT |  |
| *TS* | AAT CCG TCG AGC AGA GTT |  |
